# A Rare Case of Splenic Artery Thrombosis Provoked By Medroxyprogesterone Acetate Requiring Splenectomy

**DOI:** 10.7759/cureus.33880

**Published:** 2023-01-17

**Authors:** Asad A Haider, Raghav Bassi, Pranav Prakash, Abdullahi Hussein, Hamza Alzghoul, Muhammad Bilal, Anuoluwa Oyetoran, Uma G Iyer

**Affiliations:** 1 Internal Medicine, University of Central Florida College of Medicine, Graduate Medical Education/Hospital Corporation of America (HCA) Florida North Florida Hospital, Gainesville, USA; 2 Hematology/Oncology, University of Central Florida College of Medicine, Graduate Medical Education/Hospital Corporation of America (HCA) Florida North Florida Hospital, Gainesville, USA

**Keywords:** therapeutic anticoagulation, medication side-effects, depo-provera, spleen thrombosis, spleen infarction

## Abstract

Splenic artery thrombosis is estimated to occur in only 0.016% of hospital admissions. Hormonal contraception is known to have hypercoagulable side effects, but splenic artery thrombosis (SAT) followed by functional autosplenectomy is a very rare side effect. We report a case of a 48-year-old female with persistent SAT provoked by depot medroxyprogesterone acetate (DMPA). She initially presented with severe left lower quadrant abdominal pain, and imaging revealed an extensive thrombus in the splenic artery. She was immediately started on intravenous heparin, and her symptoms improved after a few days, at which point she was discharged on oral apixaban. Three months after discharge, the patient presented with symptoms similar to the initial presentation. Further history revealed that she received an injectable DMPA shot prior to her initial admission. Other possible causes of SAT were ruled out. On imaging, her previous thrombus had increased in size and now filled the entire splenic artery. Therefore, the patient underwent robotic splenectomy with remarkable improvement in her symptoms. This case represents a rare clinical manifestation of a hypercoagulable state induced by DMPA. We review the existing literature to explain the epidemiology, presentation, diagnosis, and treatment of SAT, and incorporate our patient’s presentation into the existing literature regarding the effect of contraception in inducing thrombotic events.

## Introduction

The spleen plays an important role in hematopoiesis and the immune system. An absent spleen increases the risk of infections and hematological abnormalities. Splenic artery thrombosis (SAT) is a rare event that occurs when the splenic artery or one of its branches becomes occluded with a thrombus, and typically presents as left-sided abdominal pain, nausea, and vomiting [1]. In severe cases, SAT can lead to functional autosplenectomy; therefore, SAT must be promptly recognized and treated. Causes of SAT include cardiogenic emboli, hypercoagulable states, infection, and medication side effects. Hormonal contraception is known to have hypercoagulable side effects, but SAT is a very rare manifestation of this class of medications. Herein, we present the case of a 48-year-old female with persistent SAT thought to be provoked by depot medroxyprogesterone acetate (DMPA).

## Case presentation

A 48-year-old female with a past medical history significant for hypertension presented to our facility with sudden severe left lower quadrant abdominal pain, nausea, and non-bloody vomiting. She denied associated symptoms of fevers, chills, diarrhea, bloody bowel movements, or dysuria. She denied any similar symptoms in the past. 

The patient reported no personal or family history of blood clots, sickle cell disease, valvular heart disease, atrial fibrillation or other arrhythmias, patent foramen ovale, malignancies, or hypercoagulable disorders. She reported no history of infection by severe acute respiratory syndrome coronavirus 2 (SARS-CoV-2). She reported no prior surgical history or hospitalizations. She denied smoking tobacco, drinking alcohol, or using any recreational drugs. She exercised regularly and reported no prolonged period of immobility. She reported normal prior mammograms, colonoscopies, and cervical cancer screenings. At this time, she informed us she was not taking any medications at home. 

**Figure 1:**
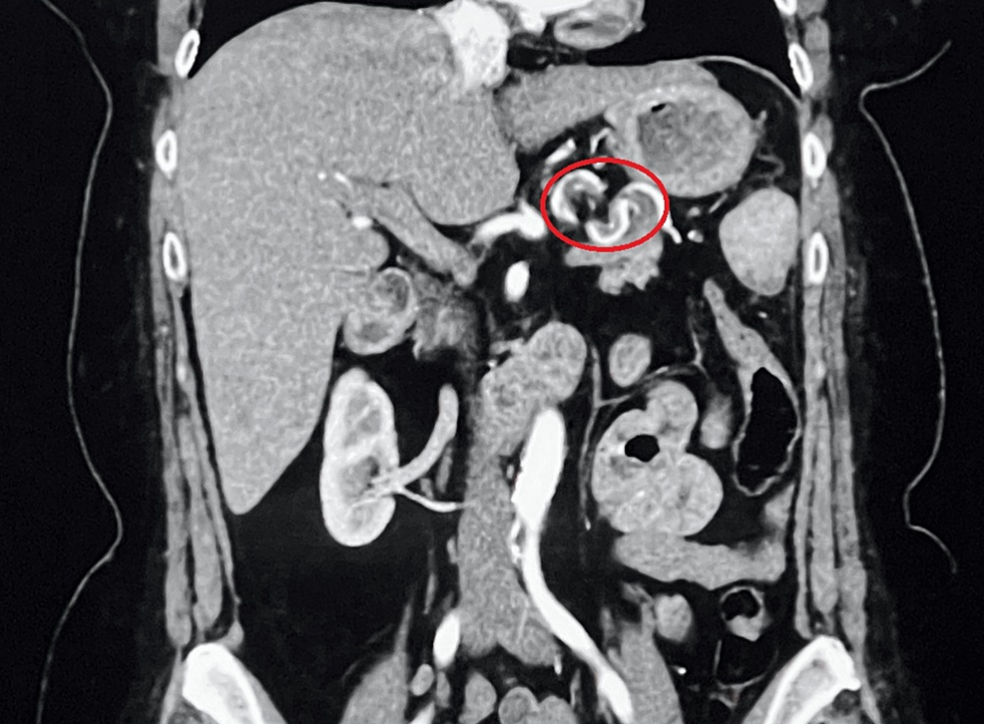
Representative image of a CTA scan of the abdomen showing an extensive filling defect in the splenic artery indicating a thrombus. Area within the red circle indicates the location of the thrombus.

In the emergency room, vital signs showed a temperature of 98.1 °F, heart rate of 86 beats/minute, respiratory rate of 16 breaths/minute, blood pressure of 147/90 mmHg, and oxygen saturation of 95% on room air. The physical exam was significant for exquisite tenderness to palpation of the left lower quadrant, but there was no guarding or rebound tenderness. The patient underwent testing for a comprehensive metabolic panel (CMP), complete blood count (CBC), troponin level, lipase level, lactic acid level, erythrocyte sedimentation rate (ESR), C-reactive protein (CRP), coagulation panel, D-dimer, urine drug screen, blood cultures, and testing for SARS-CoV-2. Significant lab abnormalities included a white blood cell (WBC) count of 11.2 thousand/mm3 (normal range 4.5-10.5 thousand/mm3), ESR of 93 mm/hr (normal range 0-15 mm/hr), CRP of 8.58 mg/dL (normal range 0.00-0.29 mg/dL), and a D-dimer of 700 ng/mL fibrinogen equivalent units (normal range 0-529 ng/mL fibrinogen equivalent units). All other lab values were within the normal range, and testing for SARS-CoV-2 was negative. An electrocardiogram showed normal sinus rhythm. A computed tomography angiography (CTA) scan of the abdomen revealed an extensive thrombus in the splenic artery, with no signs of any malignancy (Figure 1). A CTA scan of the chest showed no signs of pulmonary embolism.

The patient was immediately started on a continuous infusion of intravenous (IV) heparin. Vascular surgery was consulted to participate in the assessment and treatment of this patient alongside the internal medicine service. Vascular surgery recommended anticoagulation with apixaban rather than procedural intervention, as the patient was hemodynamically stable. A transthoracic echocardiogram was ordered, which showed no valvular stenosis, valvular regurgitation, vegetations, thrombi, or patent foramen ovale. The patient’s symptoms improved and she was discharged on day 3 of admission with instructions to take apixaban 5 mg twice a day for 6 months and follow-up outpatient with vascular surgery for monitoring of her thrombus and to undergo a hypercoagulable workup.

The patient was readmitted 3 months later with a complaint of intense left lower quadrant abdominal pain for two weeks similar in intensity to the pain on her previous admission. Unfortunately, the patient reported inconsistency in taking her prescribed apixaban due to lapses in insurance coverage. At this time, the patient recalled that she was receiving contraception in the form of injectable DMPA every 3 months. She had received a dose prior to her initial admission 3 months ago. She had completed an outpatient work-up, which showed no hereditary hypercoagulable disorder.

**Figure 2:**
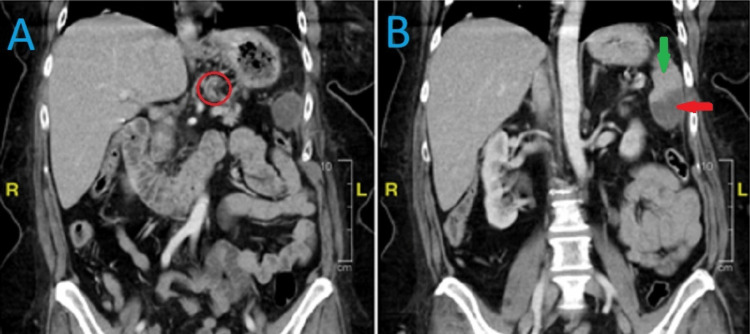
Representative image of a CT scan of the abdomen showing a filling defect indicating persistent thrombus in the splenic artery, as well as visible infarction in the spleen. (A) Area within the red circle indicates the location of the thrombus. (B) Green arrow indicates normal area of the spleen, which is hyperdense. Red arrow indicates the infarcted area of the spleen, which is hypodense.

A CMP, CBC, and coagulation panel were unremarkable. D-dimer improved to 505 ng/mL fibrinogen equivalent units (normal range 0-529 ng/mL fibrinogen equivalent units). A computed tomography (CT) scan of the abdomen showed that the previous thrombus had increased in size to fill the entire splenic artery, as well as a low-density splenic lesion noted anteriorly and inferiorly thought to represent sequela of a splenic infarct (Figure 2).

The patient was started on a continuous infusion of IV heparin. Due to the progression of the patient’s splenic infarction and lack of resolution of the thrombus with therapeutic anticoagulation, surgical consultation was sought. General surgery agreed with the need for splenectomy. Meningococcal, pneumococcal, and Hemophilus influenza vaccines were administered prior to surgery. 

**Figure 3:**
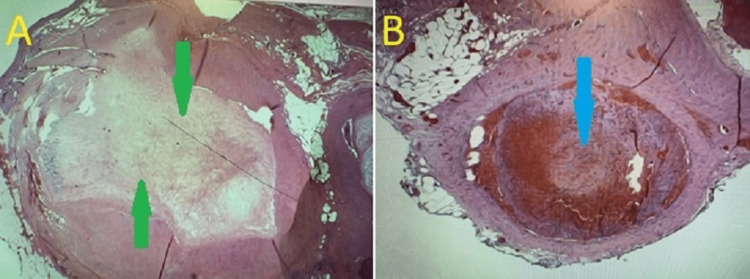
Representative image of the histology of the splenic vessels, showing thrombosis. (A) Completely thrombosed vessel, with green arrows indicating area of organized fibrosis. (B) Partially occluded vessel, with blue arrow indicating partially organizing thrombi.

**Figure 4:**
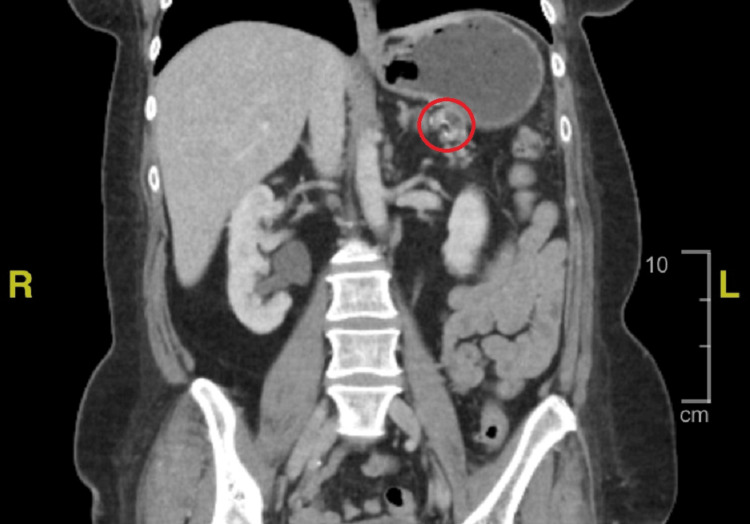
Representative image of a CT scan of the abdomen after splenectomy showing an absent spleen and a filling defect indicating thrombus in the ligated splenic artery. Area within the red circle indicates the location of the thrombus.

On day 12 of admission, the patient underwent robotic splenectomy with no complications. Part of the thrombosed splenic artery was ligated and left in the body to improve hemostasis. The specimen was sent for biopsy, which showed thrombus in the splenic vessels (Figure 3). The patient’s abdominal pain improved significantly over the next few days, and a post-splenectomy CT scan of the abdomen showed a thrombus in the ligated splenic artery and an absent spleen (Figure 4).

After a discussion among the internal medicine, hematology-oncology, and general surgery teams, the cause of the patient’s thrombus was determined to be due to the DMPA injection. Other causes of splenic infarction were excluded: the patient had no prolonged period of immobilization, no reported hypercoagulable disorder, no history of atrial fibrillation or other arrhythmias, no history of malignancy, no history of infection with SARS-CoV-2, no signs of endocarditis, no cardioembolic disease, no history of tobacco or recreational drug use, and no family history of hematologic disease. 

The patient was educated about the side effects of contraception and agreed to discontinue use of DMPA or any other hormone-based contraceptives. The patient’s health insurance was verified and she was discharged on day 15 of admission, with instructions to take apixaban indefinitely. She also agreed to follow-up outpatient with specialists from general surgery for post-operative monitoring, hematology-oncology for management of her medical anticoagulation, and obstetrics-gynecology to discuss options for non-hormonal contraception.

## Discussion

Although the exact prevalence is not known, SAT remains to be rare. However, its frequency is thought to be increasing due to increased abdominal imaging in recent years [2]. Occlusion of the splenic artery occurs due to septic or bland emboli, and typically results in splenic infarction. The most common causes of SAT are cardiogenic thromboembolism and hematologic diseases, which were ruled out in our patient. The presentation is variable, but the most common symptoms include left-sided abdominal pain (33%), fevers and chills (27%), and nausea and vomiting (22%) [3,4]. There are no consensus criteria regarding the diagnostic approach to SAT. In our patient, SAT was diagnosed with computed tomography, which is one of the most common methods used in practice [4].

The treatment of SAT typically consists of therapeutic anticoagulation and supportive care. Most patients will recover with anticoagulation alone and not require procedural intervention. The duration of anticoagulation is typically 3-6 months if provoked, and indefinite if cryptogenic. Surgical treatment is necessary in cases where symptoms persist, the clot grows significantly in size despite anticoagulation, or when complications are present (e.g. abscess, pseudocyst, autosplenectomy) [4]. Our patient’s imaging showed progression of the thrombus, as well as significant infarction of the spleen. Although resumption of anticoagulation after cessation of hormonal contraception could have been considered, it was thought that leaving the spleen intact would possibly lead to further complications. Therefore, the decision was made to proceed with a splenectomy. Our patient had no complications from surgery and her symptoms improved significantly, which demonstrates the utility of surgery in complicated cases.

Hormonal contraception is known to be associated with an increased risk of both venous and arterial thrombosis [5-7]. Although progestin-only contraceptives (POCs), such as DMPA, are thought to have a lower risk of thrombotic events than combined estrogen-progestin contraceptives, the precise magnitude of increased risk of thrombotic events with POCs is a subject of debate [8]. A study on mice has shown that DMPA can increase the risk of arterial thrombosis by increasing thrombin formation, reducing smooth muscle content, and remodeling the non-collagenous plaque matrix [9]. Although many case reports have described arterial thrombosis provoked by DMPA, no large-scale studies in humans have described a causal link between DMPA use and arterial thrombosis. To our knowledge, this is the first reported case, to date, of a SAT induced by DMPA. It is possible that the patient’s hypertension was a risk factor for the formation of thrombus, although that would likely play a minor role. Other causes of arterial thrombosis were ruled out. We argue that the temporal association between DMPA administration and the development and progression of SAT could link it to the diagnosis, especially after excluding other causes. 

DMPA, like other contraceptives, is known to cause hypercoagulability. Our case adds to the literature of a rare manifestation of this adverse effect. It is important to take a thorough medication history when evaluating risk factors for thrombosis. In some cases, patients might think an inquiry about contraceptives only refers to oral contraceptives, as this is how they are commonly described in popular culture. Therefore, it is important to ask about specific types of contraception, such as injectables or implanted devices. If a contraceptive is suspected as the cause of a thrombotic event, the contraceptive should be stopped immediately and anticoagulation should be started. In the future, large-scale studies should be undertaken to document the diverse array of clinical presentations of thrombotic events due to contraceptives. SAT remains a challenging and complex diagnosis, and it is important to be aware of its association with hormonal contraception in order to improve patient recovery times and prevent complications.

## Conclusions

Hypercoagulability is a well-known side effect of hormonal contraception, manifesting in the form of many different clinical presentations. We present a case of extensive SAT that was induced and exacerbated by DMPA while excluding other possible exacerbating factors. To our knowledge, SAT attributable to DMPA was not previously reported in the literature. Future studies should be done to further establish the relationship between hormonal contraception and the different clinical manifestations of arterial thrombosis.
